# Supplementary material for: The Proteasome Activators Blm10/PA200 Enhance the Proteasomal Degradation of N-Terminal Huntingtin
Source: Biomolecules. 2020 Nov 20;10(11):1581. doi: 10.3390/biom10111581 (PMC7699873; doi:10.3390/biom10111581)
Supplement: Supplementary file 1 [file biomolecules-10-01581-s001.pdf]

biomolecules

MDPI

Supplementary Figures

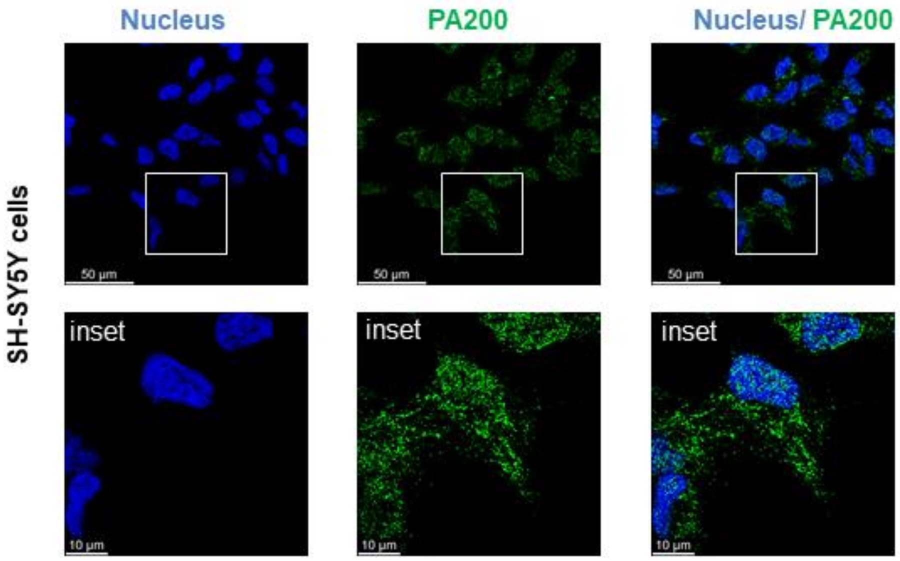

Supplementary Figure 1. Representative merged immunofluorescence confocal images showing the cellular distribution of PA200. Cells were immunolabeled with anti-PA200/Alexa Fluor 488 antibodies for PA200. DAPI was used to stain the cell nuclei.

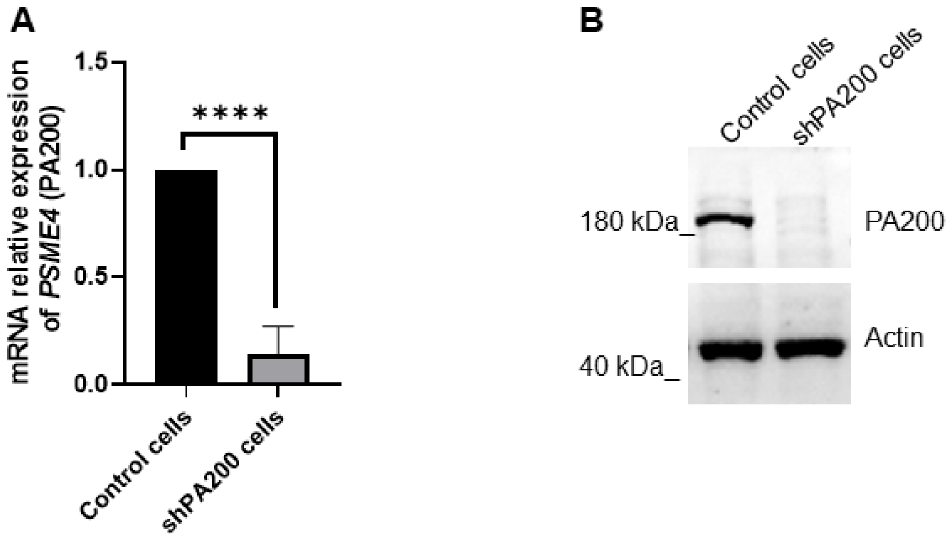

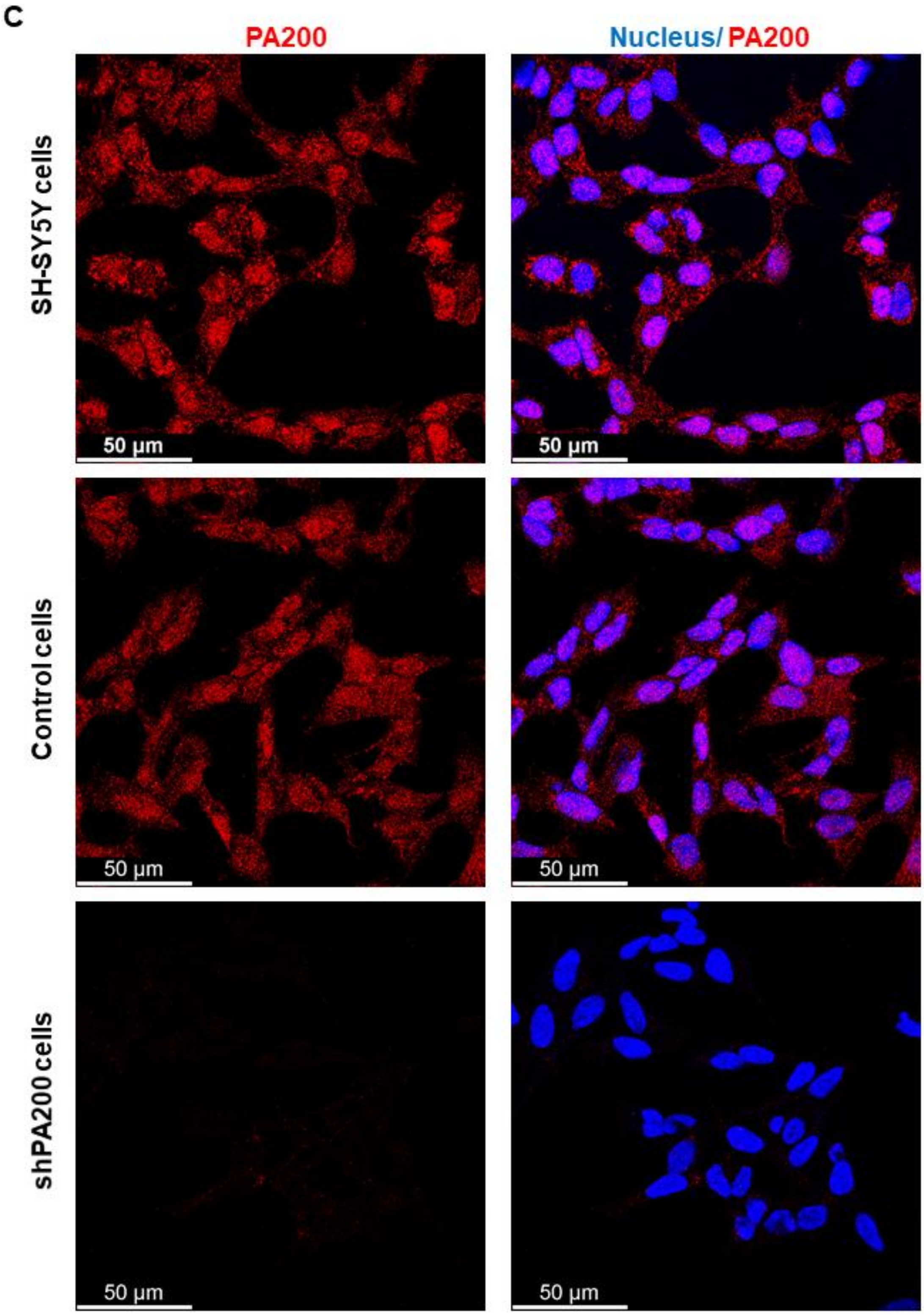

Supplementary Figure 2. Validation of PA200 depletion and the anti-PA200 antibody. The down-regulation of PA200 was analyzed by quantitative real-time PCR (A) and Western-blot (B). (A) The results of quantitative real-time PCR are presented as the mean ± SD of three independent experiments, statistical analysis was performed by unpaired student's t test using GraphPad Prism v. 8.2.1 software (**** indicates $p < 0.0001$). (B) Representative image of immunoblotting with PA200 antibody confirmed extensive depletion of PA200 in the shPA200 cell line. Equal protein amounts (30

μg) from shPA200 and control cell lines were loaded onto SDS-PAGE and followed by western blotting.

(C) Validation of the anti-PA200 antibody. Anti-PA200 antibody was used in SH SY5Y, pGIPZ-GFP scrambled control cells and in cells stably depleted for PA200 (shPA200) to test the specificity of the antibody. Cells were immunolabeled with anti-PA200/Alexa Fluor 594 for PA200. DAPI was used to stain the cell nuclei.

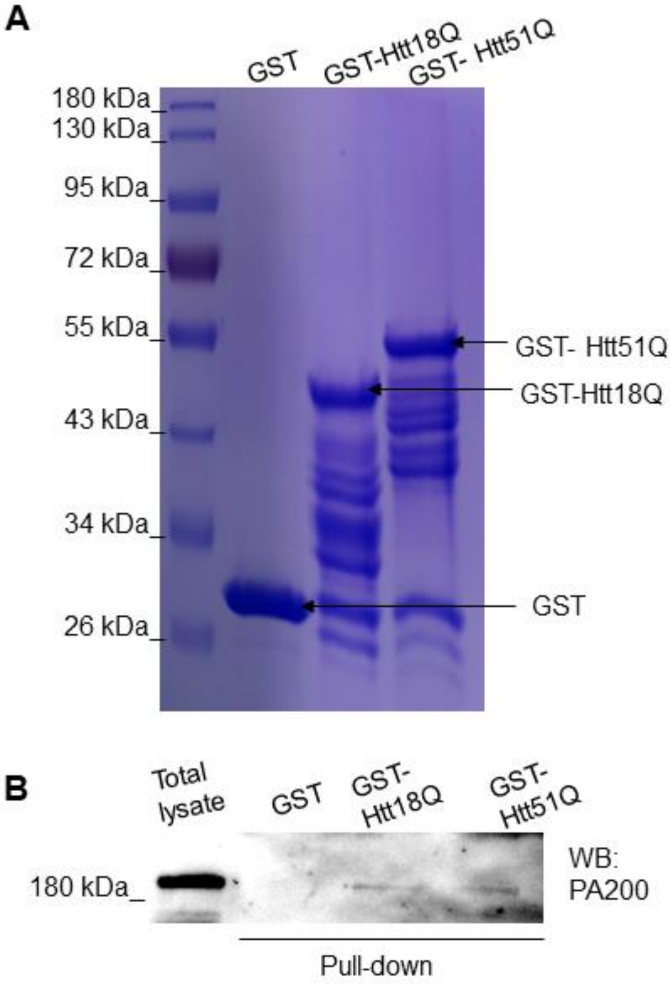

Supplementary Figure 3. PA200 binds to wt and mutant N-Htt in vitro A) Bacterially expressed glutathione S- transferase (GST), GST-Htt18Q, and GST-Htt51Q were loaded onto glutathione sepharose and purified. The efficiency of protein purification was visualized by Coomassie Blue staining.(B) GST, GST- Htt18Q, and GSTHtt51Q recombinant proteins were immobilized on GSH–Sepharose 4B and incubated with SH-SY5Y neuroblastoma cell lysate. Eluted proteins were analyzed with antibodies specific for PA200 by Western blotting.

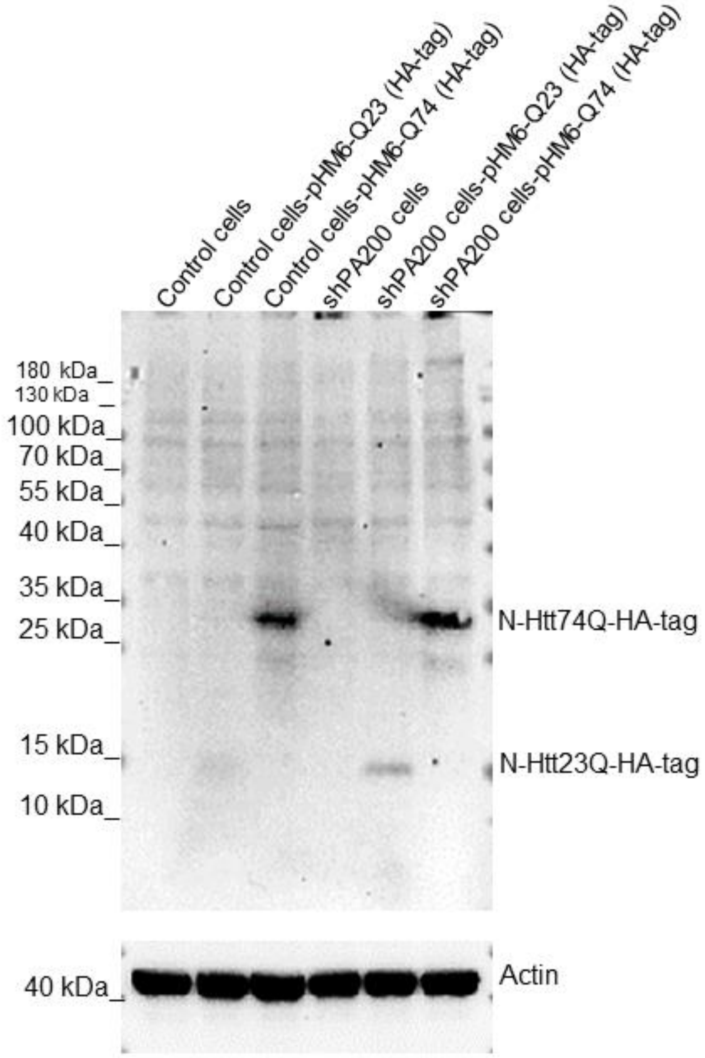

Supplementary Figure 4. Validation of normal and toxic N-Htt overexpression in control and shPA200 cells. The cells were transfected with pHM6-Q23 (HA-tag) and pHM6-Q74 (HA-tag) constructs for 48 hr. Transfected and non-transfected cells were lysed and 30 μg protein from each sample were separated by SDS-PAGE. The blots were probed with anti-HA-tag antibody and actin was used as a loading control.

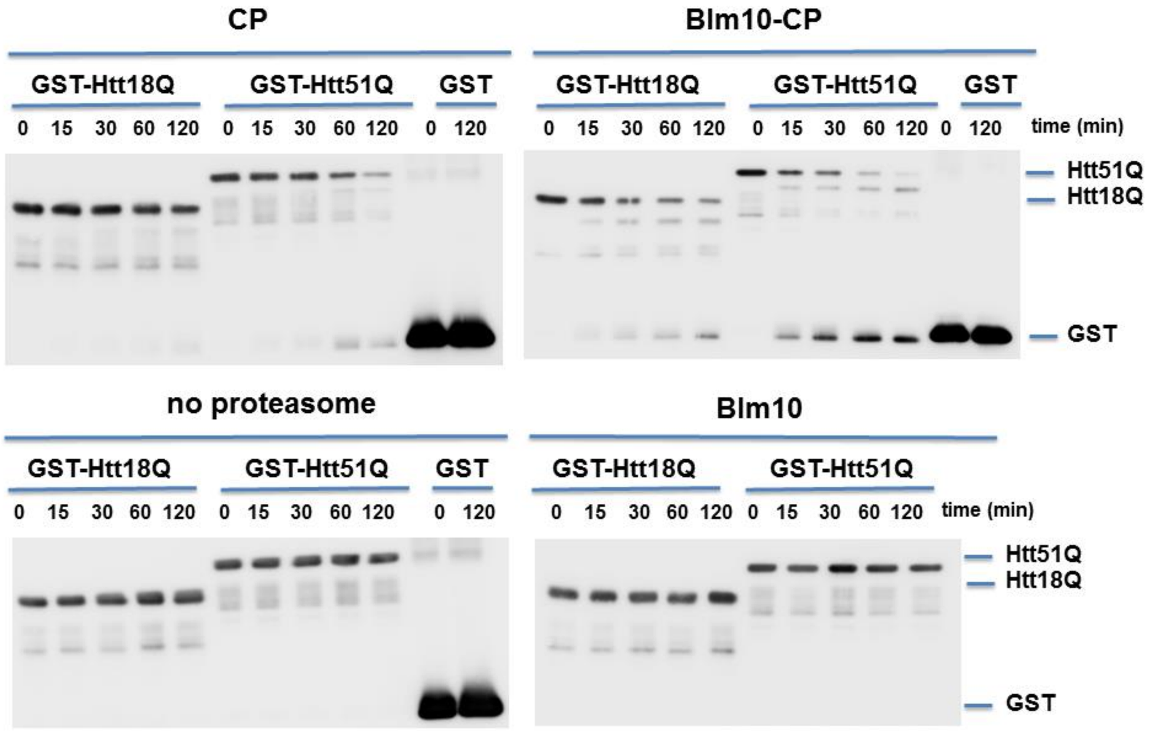

Supplementary Figure 5. The GST is not degraded by the proteasome complexes. Blm10 alone does not degrade wt and mutant N-Htt. Uncleaved GST-Htt51Q, GST-Htt18Q and GST were incubated with equal molar amounts of purified CP, Blm10-CP and Blm10. Aliquots were taken at the times indicated and separated by SDS-PAGE. Htt51Q and Htt18Q were detected by immunoblotting with a GST-specific antibody.
